# Rabphilin-3A Drives Structural Modifications of Dendritic Spines Induced by Long-Term Potentiation

**DOI:** 10.3390/cells11101616

**Published:** 2022-05-11

**Authors:** Luca Franchini, Jennifer Stanic, Marta Barzasi, Elisa Zianni, Daniela Mauceri, Monica Diluca, Fabrizio Gardoni

**Affiliations:** 1 Department of Pharmacological and Biomolecular Sciences, University of Milan, 20133 Milan, Italy; luca.franchini@unimi.it (L.F.); jennifer.stanic@unimi.it (J.S.); marta.barzasi@studenti.unimi.it (M.B.); elisa.zianni@unimi.it (E.Z.); monica.diluca@unimi.it (M.D.); 2 Department of Neurobiology, Interdisciplinary Center for Neurosciences (IZN), Heidelberg University, INF 366, 69120 Heidelberg, Germany; mauceri@nbio.uni-heidelberg.de

**Keywords:** long-term potentiation, dendritic spines, NMDA receptors, AMPA receptors

## Abstract

The interaction of Rabphilin-3A (Rph3A) with the NMDA receptor (NMDAR) in hippocampal neurons plays a pivotal role in the synaptic retention of this receptor. The formation of a Rph3A/NMDAR complex is needed for the induction of long-term potentiation and NMDAR-dependent hippocampal behaviors, such as spatial learning. Moreover, Rph3A can also interact with AMPA receptors (AMPARs) through the formation of a complex with myosin Va. Here, we used a confocal imaging approach to show that Rph3A overexpression in primary hippocampal neuronal cultures is sufficient to promote increased dendritic spine density. This morphological event is correlated with an increase in GluN2A-containing NMDARs at synaptic membranes and a decrease in the surface levels of GluA1-containing AMPARs. These molecular and morphological modifications of dendritic spines are sufficient to occlude the spine formation induced by long-term potentiation, but do not prevent the spine loss induced by long-term depression. Overall, our results demonstrate a key role for Rph3A in the modulation of structural synaptic plasticity at hippocampal synapses that correlates with its interactions with both NMDARs and AMPARs.

## 1. Introduction

The remodeling of neuronal circuits is strictly correlated to activity-dependent modifications of dendritic spine morphology and changes in the number and strength of synaptic contacts. Overall, the structural plasticity of excitatory synapses represents the cellular correlates of learning and memory processes [1].

The induction of activity-dependent synaptic plasticity and the subsequent morphological modifications of dendritic spines are complex events. In addition to changes in the structure of pre-existing spines, the induction of long-term potentiation (LTP) and long-term depression (LTD) are also associated with the formation of new spines and the loss of pre-existing spines, respectively [2]. These morphological modifications require a coordinated involvement of postsynaptic glutamate receptors. The role of NMDA-type glutamate receptors (NMDAR) has been widely discussed, and several studies have addressed the contribution of the specific regulatory GluN2-type subunits of NMDARs in these events [3,4]. In particular, synaptic retention of the GluN2A subunit has been intensively studied, as GluN2A-containing NMDARs display specific channel properties and calcium dynamics, and lead to the activation of specific intracellular pathways that intrinsically direct plasticity signaling. It is also well known that the GluN2A intracellular C-terminal domain (CTD) interacts with a variety of synaptic proteins with different functions, and these protein–protein interactions play a key role for several properties of NMDARs, ranging from the synaptic retention of the receptor to its role in LTP induction [4]. However, the relevance of specific GluN2A interactors at the CTD in driving the molecular and morphological modifications of dendritic spines associated with the induction of synaptic plasticity is not fully understood.

Rabphilin-3A (Rph3A) is a binding protein of the GluN2A CTD that is specifically associated with the synaptic-retained NMDARs [5,6]. Rph3A was first identified as a Rab3A binding partner at presynaptic vesicles [7], but it is able to bind several other proteins, including the MAGUK protein CASK [8], synaptotagmin-1 [9], SNAP-25 [10], Arf6 [11] and myosin Va (MyoVa) [12], localized both in the pre- or post-synaptic compartment. In particular, Rph3A at dendritic spines forms a molecular complex with GluN2A and PSD-95, which is required for NMDAR synaptic retention [5,6]. Notably, Rph3A silencing or disruption of interaction of Rph3A with GluN2A or PSD-95 leads to reduced NMDAR synaptic retention and loss of dendritic spines [5,6]. A careful confocal and electron microscopy analysis showed that Rph3A is not present in all dendritic spines in an even manner, but rather, under basal conditions, it is localized in approximately 50% of hippocampal spines [6]. Importantly, Rph3A positive (Rph3A+) spines have an increased spine head area and postsynaptic density length and thickness, suggesting a higher stability of neuronal transmission through these Rph3A-enriched connections [6]. 

The interactions of Rph3A with NMDARs and other synaptic partners are positively regulated by Ca^2+^ influx and inositol triphosphate (IP_3_) levels at synapses [5,9,10,13,14]. Interestingly, Rph3A, through both its C2A and C2B domains, binds IP_3_ in a Ca^2+^-dependent manner. In particular, Ca^2+^ induces a conformational rearrangement of the CBL3 Rph3A loop, which is involved in IP_3_ binding, dramatically increasing the formation of Rph3A complexes with GluN2A and other interactors. Notably, LTP induction leads to an increased number of Rph3A+ spines and to an augmented NMDAR synaptic retention, thus indicating that activity-dependent plasticity promotes the postsynaptic localization of the protein [6]. In addition, Rph3A silencing or disruption of the Rph3A/NMDAR complex not only blocks GluN2A accumulation at postsynaptic membranes, but also prevents the induction of LTP and the formation of new spines. Accordingly, the treatment of mice with either Rph3A silencing or peptides disrupting the Rph3A/NMDAR complex impairs the acquisition of spatial memories [6]. 

Recently, Rph3A was also indicated as novel target for the maintenance of cognition in old age [15]. Conversely, aberrant Rph3A expression and interactions with GluN2A-containing NMDARs were observed in models of pathological synaptic plasticity, such as parkinsonian rats displaying a dyskinetic profile [16]. The treatment of dyskinetic animals with a Rph3A/GluN2A interfering peptide significantly reduced their abnormal motor behavior [16].

Here, we show that Rph3A overexpression in primary hippocampal neuronal cultures is sufficient to induce molecular modifications both at AMPARs and NMDARs, leading to dendritic spine formation and the occlusion of LTP-induced modifications of spine density.

## 2. Materials and Methods

### 2.1. Cell Cultures

Hippocampal primary neuronal cultures were prepared from embryonic day 18–19 (E18–E19) rat hippocampi, as previously reported [17]. Neurons were transfected on day in vitro 7 (DIV7) through the calcium-phosphate method or infected with adeno-associated virus serotype 9 (AAV9) on DIV3. Neurons were used for the various experiment on DIV16.

### 2.2. Cell Fractionation and Postsynaptic Density Purification

Triton insoluble fractions (TIF) were isolated from rat hippocampal primary cultures. TIF is a fraction highly enriched in postsynaptic density proteins [18]. The samples were homogenized at 4 °C in an ice-cold buffer containing 0.32 M sucrose, 0.1 mM phenylmethylulfonyl fluoride (PMSF), 1 mM HEPES, 1 mM MgCl, and 1 mM NaF, supplemented with protease inhibitors (Complete™, Sigma-Aldrich, St. Louis, MI, USA) and phosphatase inhibitors (PhosSTOP™, Sigma-Aldrich). The homogenate was centrifuged at 13,000× *g* for 15 min at 4 °C, and the resulting pellet representing the P2 fraction was resuspended in 0.5% Triton-X-100 and 75 mM KCl for 15 min at 4 °C. The samples were then centrifuged at 100,000× *g* for 1 h at 4 °C, and the pellets obtained that represent the TIF were resuspended in 20 mM HEPES.

### 2.3. Immunocytochemistry (ICC)

For colocalization and morphological studies, transfected hippocampal neurons were fixed for 10 min at room temperature (RT) in 4% paraformaldehyde (PFA) and 4% sucrose in Dulbecco’s phosphate buffered saline (PBS). Coverslips were then washed with PBS, permeabilized with 0.1% Triton X-100 in PBS for 15 min at RT, and blocked for 30 min at RT with 5% bovine serum albumin (BSA) in PBS. Cells were then incubated with primary antibodies in 3% BSA-PBS overnight at 4 °C in a humidified chamber. After washes with PBS, the cells were incubated with the fluorophore-conjugated secondary antibodies in 3% BSA-PBS for 1 h at RT in a humidified chamber protected from light. The incubation was followed by washes with PBS and mounting onto glass slides using a Fluoroshield mounting medium (Sigma-Aldrich). For the surface staining assays, cells were not permeabilized and were labeled with primary antibody for extracellular epitopes overnight at 4 °C. The coverslips were then washed in PBS, and a secondary antibody conjugated to Alexa Fluor dye was used.

### 2.4. Western Blot (WB)

Protein samples were separated using a denaturing sodium dodecyl-sulfate polyacrylamide gel electrophoresis (SDS-PAGE) followed by Western blotting onto nitrocellulose membranes. The membranes were incubated for 1 h at RT in blocking solution (I-block, Tris-buffered saline [TBS] 1X, 20% Tween 20) on a shaker. The membranes were then incubated with the specific primary antibodies in blocking solution overnight at 4 °C and, the following day, after three washes with TBS and 0.1% Tween 20 (TBSt), they were incubated with the corresponding horseradish peroxidase (HRP)-conjugated secondary antibody in blocking solution for 1 h at RT. After washing with TBSt, the membranes were developed using electrochemiluminescence (ECL) reagents (Bio-Rad, Hercules, CA, USA). Finally, the membranes were scanned using a Chemidoc (Bio-Rad) with Image Lab software (Bio-Rad). The bands were quantified by means of computer-assisted imaging (Image Lab, Bio-Rad). The levels of the proteins were expressed as relative optical density (OD) measurements, normalized to tubulin, and then expressed as percentage of the control mean.

### 2.5. Co-Immunoprecipitation Assay

Proteins from rat hippocampal tissues were incubated on a wheel overnight at 4 °C in RIA buffer 1× (200 mM NaCl, 10 mM EDTA, 10 mM Na_2_HPO_4_, 0.5% NP-40) plus 0.1% SDS and primary antibodies. The day after, magnetic beads A (Bio-Rad #1614013) were washed in PBS, resuspended in RIA 1×, added to the samples, and incubated at RT on a wheel for 1 h. The beads were then sedimented and the supernatant was discarded. The beads were washed three times with RIA 1× + 0.1% SDS mixed with loading buffer (3×), and boiled for 10 min at 96 °C for WB procedures.

### 2.6. Chemical LTP (cLTP) and Chemical LTD (cLTD)

cLTP was induced on DIV16 using a previously validated protocol [6,19,20,21]. The neurons were incubated in artificial cerebrospinal fluid (ACSF, 125 mM NaCl, 25 mM KCl, 2 mM CaCl_2_, 33 mM glucose and 25 mM HEPES) + 1 mM MgCl_2_ for 30 min at 37 °C. cLTP induction was then performed in ACSF without MgCl_2_, plus 50 µM forskolin (Tocris, Bristol, UK), 0.1 µM rolipram (Tocris), and 100 µM picrotoxin (Tocris) for 16 min. Control groups were kept in normal ACSF. Next, the cells were incubated in ACSF with MgCl_2_. To induce cLTD, neuronal cultures were first incubated in ACSF for 30 min, and then stimulated with 50 µM NMDA (Sigma-Aldrich) in ACSF [21,22]. After 10 min of stimulation, the NMDA solution was replaced with regular ACSF for 20 min.

### 2.7. SUnSET Assay

Neurons were treated with the cLTP protocol, and a SUnSET experiment was performed as previously described [23]. Puromycin (10 µm) was added during cLTP in the last 5′ resting min. The coverslips were then immediately washed with PBS supplemented with calcium and magnesium, and fixed for the staining of Rph3A, puromycin and GFP. For each neuron, the mean of the integrated density of the puromycin signal in Rph3A+ spines was compared to Rph3A- spines.

### 2.8. Confocal Imaging

Images were taken using an inverted LSM900 confocal microscope (Zeiss, Ginner, Germany) with a 63× objective, and were analyzed using ImageJ software. Cells were chosen randomly for quantification from different coverslips from independent experiments and images were acquired using the same settings/laser power. For spine morphology, Z-stack images were taken with a Z-step of 0.35 µm. Analysis of dendritic spine morphology was performed with ImageJ. For each dendritic spine, length, head, and neck width were measured, which was used to classify dendritic spines into three categories (thin, stubby and mushroom) [5,24,25]. In particular, the length and the ratio between the width of head and the width of neck (Wh/Wn) were used as parameters for the classification as follows: protrusions having a length of more than 3 μm were considered as filopodia, the others as spines; spines with a Wh/Wn ratio bigger than 1.7 were considered mushrooms; spines with a Wh/Wn ratio smaller than 1.7 were divided into the categories of stubby, if shorter than 1 μm, and thin if longer than 1 μm. Protrusions with length over 5 μm were excluded from the analysis. 

Live imaging of GFP or pH-sensitive superecliptic pHluorin (SEP)-GluA1/RFP-Rph3A was conducted using an inverted LSM900 confocal microscope (Zeiss) in ACSF at 37 °C in an atmosphere of 5% CO_2_. Z-stack images with a Z-step of 0.40 µm were acquired over a period of 60 to 90 min (every 5 min). To examine the population of surface SEP-GluA1, we used a low pH solution adjusted to pH 5.4, which quenched all the fluorescence, indicating that SEP allows for the specific visualization of surface receptors. Fluorescence intensity was measured using ImageJ only at selected regions of interest (ROI; i.e., spines), and corrected for background noise. Δ*F*/*F*0 was then calculated and plotted. Spines and filopodia were counted and spine head width was measured with ImageJ.

### 2.9. Colocalization in Airyscan Modality

Images were taken in super-resolution modality using the Airyscan mode on a LSM900 confocal microscope (Zeiss) with a 63× objective at a 0.04 μm pixel size. Colocalization analysis was performed using Zen software (Zeiss).

### 2.10. Antibodies

The following primary antibodies were used: rabbit anti-Rph3A (WB 1:1000, #118003, Synaptic Systems, Gottingen, Germany), rabbit anti-GluA1 (WB 1:1000, #31232, Abcam, Cambridge, UK), monoclonal rabbit anti-phosphoSer845GluA1 (WB 1:1000, #ab76321, Abcam), rabbit anti-GluN2A (WB 1:1000, #M264, Sigma-Aldrich), rabbit anti-MyoVA (ICC 1:300, WB 1:1000, #3402S, Cell Signaling, Danvers, MA, USA), monoclonal mouse anti-tubulin (WB 1:30000, #T9026, Sigma-Aldrich), monoclonal mouse anti-puromycin (ICC, 1:200, #MABE343, Sigma-Aldrich), mouse anti-RFP (ICC 1:500, #OAEA00012, Aviva, London, UK), chicken anti-GFP (ICC 1:300, #AB16901, Millipore, Burlington, MA, USA), rabbit anti-N-term-GluN2A (ICC 1:100, #480031, Invitrogen, Waltham, MA, USA), mouse anti-GluA1 (ICC 1:100, #75-327, Millipore), and mouse anti-PSD-95 (ICC 1:1000, #192757, Abcam). The following secondary antibodies were used for WB analysis: goat anti-rabbit HRP and goat anti-mouse HRP (#1706515 and #1706516, respectively, Bio-Rad). For ICC, the following secondary antibodies were used: goat anti-mouse Alexa Fluor 405 (#A31553, Life Technologies, Carlsbad, CA, USA), goat anti-rabbit Alexa Fluor 568 (Life Technologies), donkey anti-rabbit Alexa Fluor 555 (#A31572, Life Technologies), donkey anti-rabbit Alexa Fluor 647 (#A31573, Life Technologies), goat anti-chicken Alexa Fluor 488 (#A11039, Life Technologies), goat anti-mouse Alexa Fluor 555 (#A21424, Life Technologies), goat anti-chicken Alexa Fluor 488 (#13C0523, Immunological Sciences, Rome, Italy), goat anti-rabbit Alexa Fluor 488 (#A21206, Life Technologies), goat anti-rabbit Alexa Fluor 647 (#A21245, Life Technologies), goat anti-mouse Alexa Fluor 488 (#411029, Life Technologies), and goat anti-mouse Alexa Fluor 647 (#A21235, Life Technologies).

### 2.11. Reagents

Forskolin (#1099/10), rolipram (#0905), and picrotoxin (#1128) were purchased from Tocris. NMDA (#M3262) and puromycin (#P8833) were purchased from Sigma-Aldrich. The GFP plasmid was kindly provided by Dr M. Passafaro. The hSyn-RFP-WPRE, hSyn-RFPRph3A-WPRE, and CMV-SEP-GluA1 plasmids were purchased from Addgene. AAV9-hSyn-RFPRph3A-WPRE, and its control AAV9-hSyn-RFP-WPRE was produced and purchased from ICGEB (Trieste, Italy).

### 2.12. Data Presentation and Statistical Analysis

All of the group values are expressed as mean ± SEM. Normality was examined using D’Agostino–Pearson or Shapiro–Wilk tests. Comparisons between groups were performed using the following tests as appropriate: two-tailed unpaired Student’s t-test, Mann–Whitney U test, one-way analysis of variance (ANOVA) or two-way ANOVA, followed by Tukey, Sidak or Dunnett post-hoc tests. Outliers were identified with the ROUT method (Q = 5%). Significance was defined as *p* < 0.05. All statistical analyses were performed using the GraphPad Prism statistical package (GraphPad software). Sample sizes for the specific types of experiments conducted are similar to those generally employed in the field. When appropriate, experiments were performed under blind conditions.

## 3. Results

### 3.1. Rph3A Overexpression Promotes Dendritic Spine Formation

**Figure 1:**
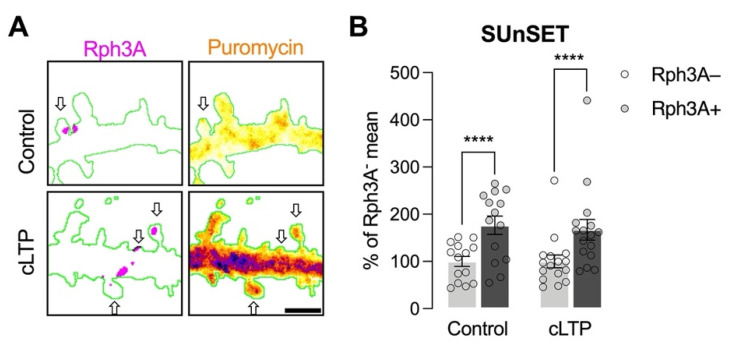
Analysis of the role of Rph3A in protein translation at dendritic spines. (**A**): Confocal images of dendritic spines shown by a fluorescent filler (GFP, green), Rph3A staining (red), and puromycin (rainbow), as well as a merged image (right panels). Arrows point out Rph3A+ spines. Scale bar: 1 µm. (**B**): Bar graph representation of mean ± SEM puromycin levels in Rph3A− (light grey) and Rph3A+ (dark grey) spines under control conditions or after cLTP induction. The data are expressed as a percentage of Rph3A− mean puromycin levels. Rph3A+ control vs. Rph3A− control: Paired *t*-test; Rph3A+ cLTP vs. Rph3A−, cLTP: Wilcoxon test. **** *p* < 0.0001. Dots represent single values.

More active dendritic spines require a higher turnover of protein translation compared to less active ones [26,27]. Similarly, tagged synapses are able to capture more plasticity related proteins upon synaptic stimulation, which are necessary for the long-term maintenance of synaptic plasticity events [28,29]. Based on the observation that Rph3A is localized in approximately 50% of hippocampal spines [6], we investigated whether the presence of Rph3A in spines could represent more efficient sites of glutamatergic neurotransmission. To this end, we performed a SUnSET experiment to detect the levels of newly synthetized proteins in Rph3A+ and Rph3A- spines, both in the resting state and after cLTP induction. As shown in Figure 1A,B, Rph3A+ spines display a higher level of newly synthetized proteins both under resting conditions (*n* = 14 neurons; *p* < 0.0001) and after cLTP (*n* = 16 neurons; *p* < 0.0001), indicating an augmented turnover of protein translation in Rph3A+ spines. 

**Figure 2:**
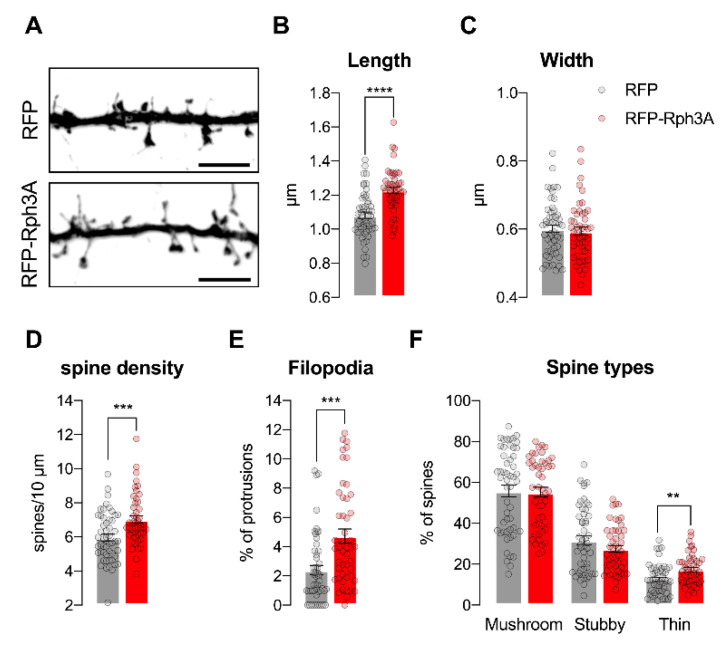
Rph3A overexpression promotes dendritic spine formation. (**A**): Confocal images of GFP filler in the dendritic spines of RFP (**up**) or RFP-Rph3A (**down**) transfected neurons. Scale bar: 5 µm. (**B**–**F**): Bar graph representation of mean ± SEM spine length (**B**), spine head width (**C**), spine density (**D**), filopodia proportions (**E**), and spine types percentage (**F**) in RFP (grey) or RFP-Rph3A (red) transfected neurons. Unpaired *t*-test (length, width, spine types), Mann–Whitney U test (spine density, filopodia). ** *p* < 0.01; *** *p* < 0.001; **** *p* < 0.0001. Dots represent single values.

We previously reported that Rph3A activity at dendritic spines is needed to prevent spine loss under resting conditions [5]. Starting from these considerations, we transfected Rph3A fused to a fluorescent tag (RFP) into primary hippocampal neurons at DIV7 and assessed the effect of its overexpression on the morphological parameters of dendritic spines in vitro at DIV16. As shown in Figure 2A–F, RFP-Rph3A transfected neurons display a significant increase in the density of both dendritic spines (*n* = 48–52 neurons; *p* < 0.001) and filopodia-like structures (*n* = 48–52 neurons; *p* < 0.001) associated with alterations in spine morphology. In particular, the dendritic spines of Rph3A-transfected neurons are longer (*n* = 48–52 neurons; *p* < 0.0001), with no difference in head width (*n* = 48–52 neurons; *p* = 0.6457). An accurate classification of dendritic spines reveals that the thin spine percentage is significantly increased in RFP-Rph3A neurons (*n* = 48–52 neurons; *p* < 0.01), without alterations in the other spine subtypes.

### 3.2. Live-Imaging of Dendritic Spines: Effects of Rph3A Overexpression

**Figure 3:**
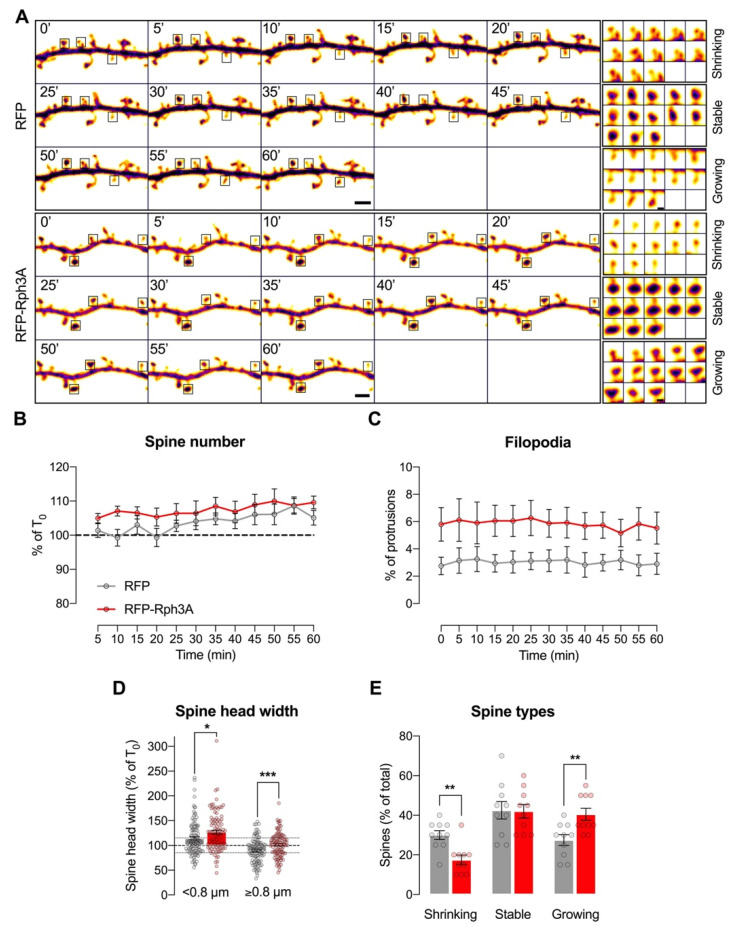
Rph3A overexpression in live-imaging of dendritic spines. (**A**): Confocal time lapse images of GFP filler in the dendritic spines of RFP (**up**) or RFP-Rph3A (**down**) transfected neurons. Scale bar: 2 µm. Inserts show examples of shrinking, stable and growing spines for each condition. Scale bar: 0.5 µm. (**B**): XY graph representing mean ± SEM spine number as a percentage of T_0_ (0 min) in RFP (grey) or RFP-Rph3A (red) transfected neurons over time. (**C**): XY graph representing mean ± SEM filopodia as a percentage of total protrusions in RFP (grey) or RFP-Rph3A (red) transfected neurons over time. (**D**): Bar graph representing mean ± SEM spine head width as a percentage of T_0_ of small spines (<0.8 µm) and big spines (≥0.8 µm) in RFP (grey) or RFP-Rph3A (red) transfected neurons at T_60_ (60 min), Mann–Whitney U test (<0.8 µm), unpaired t-test (≥0.8 µm). Dots represent single values. (**E**): Bar graph representing mean ± SEM percentage of different spine types (shrinking, stable or growing) at T_60_, Mann–Whitney U test (shrinking), unpaired *t*-test (growing). * *p* < 0.05; ** *p* < 0.01; *** *p* < 0.001. Dots represent single values.

We then moved to evaluate the morphological behavior of spines in basal conditions through confocal live-imaging. RFP-Rph3A transfected neurons display stable spine (Figure 3A,B) and filopodia (Figure 3A,C) numbers similar to RFP-transfected control neurons (*n* = 10 neurons) within a 60 min timeframe. However, when focusing on spine head width, Rph3A overexpressing neurons show an increased tendency for smaller spines (head width less than 0.8 µm) to grow within the timeframe compared to control neurons (Figure 3A,D; *n* = 97–102 spines; *p* < 0.05). Conversely, bigger spines (head width equal to or more than 0.8 µm) are significantly more stable in Rph3A overexpressing neurons than in control neurons (Figure 3A,D; *n* = 97–103 spines; *p* < 0.001). 

Moreover, when organizing the different spines according to the percentage of their starting head width (T_0_) into 3 categories (shrinking, stable or growing), Rph3A overexpressing neurons are characterized by a significant decrease of the proportion of shrinking spines (*n* = 10; *p* < 0.01) to the benefit of growing spines (*n* = 10 neurons; *p* < 0.01; Figure 3A,E).

### 3.3. Rph3A Overexpression Is Sufficient for Synaptic and Surface Stabilization of GluN2A-Containing NMDARs

**Figure 4:**
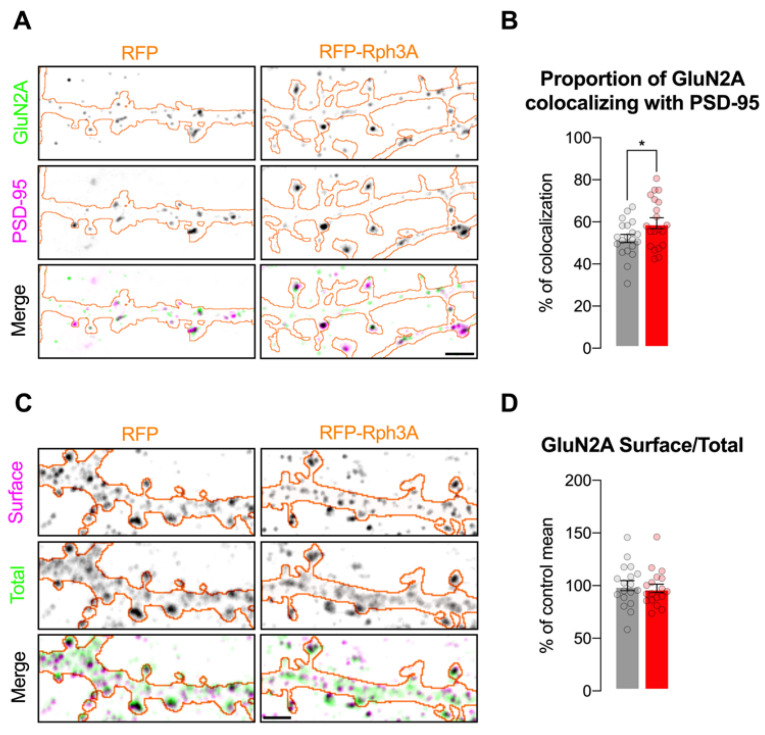
Rph3A overexpression is sufficient for synaptic and surface stabilization of GluN2A-containing NMDARs. (**A**): Airyscan confocal images of GluN2A (green) and PSD-95 (magenta) staining in RFP (**left**) or RFP-Rph3A (**right**) transfected neurons (orange outline). Scale bar: 2 µm. (**B**): Bar graph representing mean ± SEM of percentage of GluN2A colocalizing with PSD-95, unpaired *t*-test. (**C**): Confocal images of surface (magenta) and total GluN2A (green) staining in RFP (left) or RFP-Rph3A (right) transfected neurons (orange outline). Scale bar: 2 µm. (**D**): Bar graph representing mean ± SEM of percentage of control mean of GluN2A surface/total ratio, Mann–Whitney U test. * *p* < 0.05. Dots represent single values.

We previously demonstrated that disrupting the interaction of Rph3A with the GluN2A subunit of NMDARs leads to a decreased GluN2A synaptic retention that is associated with a reduced dendritic spine density [5]. Starting from these data, here, we evaluated whether the increased spine density observed in neurons overexpressing Rph3A is associated with molecular alterations in GluN2A-containing NMDAR levels. Airyscan confocal microscopy shows that Rph3A overexpression significantly increases the proportion of GluN2A stained pixels colocalizing with the post-synaptic marker PSD-95, when compared to control neurons (Figure 4A,B; *n* = 20 neurons; *p* < 0.05). 

Conversely, the overall surface/total ratio of GluN2A staining along dendrites in Rph3A overexpressing neurons analyzed with confocal microscopy did not significantly differ compared to controls (Figure 4C,D; *n* = 18–19 neurons, *p* = 0.5418). To exclude putative effects of Rph3A overexpression on the GluN2A protein levels, WB analysis was performed on neuronal lysates from cultures infected with AAV9-hSyn-RFPRph3A-WPRE and its control AAV9-hSyn-RFP-WPRE (Figure S1). Viral infection promoted the expression of RFP-Rph3A protein levels in the absence of any alterations in the GluN2A (*n* = 9 cultures, *p* = 0.6798; data not shown) or GluN2B subunits (*n* = 8 cultures, *p* = 0.7265; data not shown) of NMDARs. Overall, these results suggest an increased presence of GluN2A-containing NMDARs localized at PSD-95 positive synaptic sites in neurons overexpressing Rph3A.

### 3.4. Rph3A Overexpression Alters GluA1-AMPARs Phosphorylation and Surface Levels 

**Figure 5:**
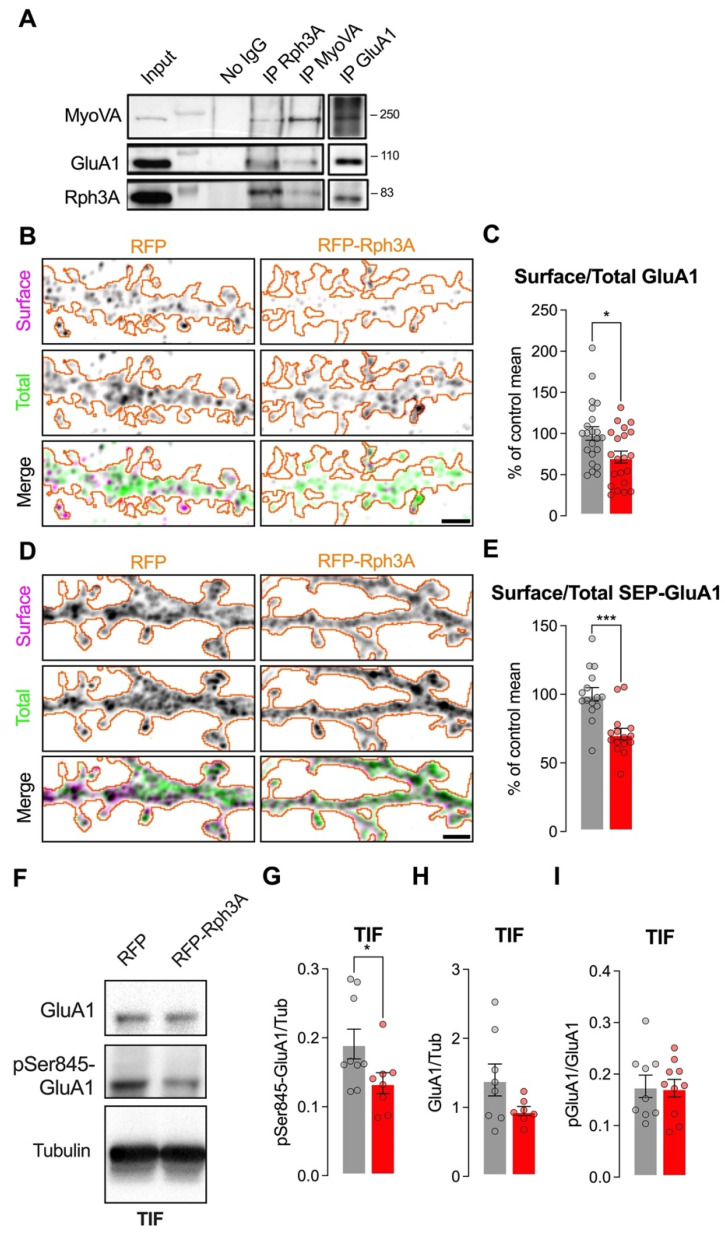
Rph3A overexpression alters GluA1-AMPAR phosphorylation and surface levels. (**A**): Western blot images of Rph3A/MyoVA/GluA1 co-immunoprecipitation from hippocampal lysates. (**B**): Confocal images of surface (magenta) and total GluA1 (green) staining in RFP (**left**) or RFP-Rph3A (**right**) transfected neurons (orange outline). Scale bar: 2 µm. (**C**): Bar graph representing mean ± SEM of percentage of control mean of GluA1 surface/total ratio, unpaired *t*-test. (**D**): Confocal images of surface (magenta) and total SEP-GluA1 (green) staining in RFP (**left**) or RFP-Rph3A (**right**) transfected neurons (orange outline). Scale bar: 2 µm. (**E**): Bar graph representing mean ± SEM of percentage of control mean of SEP-GluA1 surface/total ratio, unpaired *t*-test. (**F**): Western blot images of pSer845-GluA1, GluA1 and tubulin from the triton insoluble fraction (TIF) of hippocampal cultured neurons infected with AAV9-hSyn-RFP-WPRE or AAV9-hSyn-RFPRph3A-WPRE. (**G**): Bar graph representing mean ± SEM of the pSer845-GluA1/tubulin staining ratio from the TIF of hippocampal cultured neurons infected with AAV9-hSyn-RFP-WPRE or AAV9-hSyn-RFPRph3A-WPRE, Mann–Whitney U test. (**H**): Bar graph representing mean ± SEM of the GluA1/tubulin staining ratio from the TIF of hippocampal cultured neurons infected with AAV9-hSyn-RFP-WPRE or AAV9-hSyn-RFPRph3A-WPRE, Mann–Whitney U test. (**I**): Bar graph representing mean ± SEM of the pSer845-GluA1/GluA1 staining ratio from the TIF of hippocampal cultured neurons infected with AAV9-hSyn-RFP-WPRE or AAV9-hSyn-RFPRph3A-WPRE, unpaired *t*-test. * *p* < 0.05, *** *p* < 0.001; Dots represent single values.

Synaptic connections are strengthened upon LTP by increased insertion of GluA1-containing AMPARs at postsynaptic membranes [30], and this process is promoted in part by MyoVA activity [31]. A literature screening of Rph3A protein partners showed that MyoVA interacts with the C2 domain of Rph3A and mediates vesicle exocytosis [12]. We therefore probed the existence of a trimeric complex composed of Rph3A/MyoVA/GluA1 in the brain, and asked whether modulation of Rph3A expression could influence not only NMDARs, but also AMPAR localization in dendritic spines. For this purpose, we first immunoprecipitated Rph3A, GluA1, and MyoVA from hippocampal lysates, and, strikingly, we always detected the three proteins in the immunoprecipitated materials (Figure 5A), indicating the existence of a Rph3A/MyoVA/GluA1 protein complex in the hippocampus. 

Notably, Rph3A overexpression induced a significant reduction in GluA1 surface levels, as analyzed by confocal imaging of the endogenous GluA1 surface/total staining ratio (*n* = 21–22 neurons; *p* < 0.05; Figure 5B,C). The same results were obtained by using SEP, whose fluorescence is quenched at a low pH. Indeed, analysis of GluA1 surface levels by using SEP-tagged GluA1 indicated a decreased staining ratio in neurons overexpressing RFP-Rph3A compared to controls (*n* = 15 neurons; *p* < 0.001; Figure 5D,E). 

It is well known that GluA1 retention at membranes is strictly correlated with its phosphorylation at Ser845 (pSer845-GluA1) [30]. Thus, we evaluated whether the reduction in surface GluA1 in neurons overexpressing Rph3A is associated with an altered phosphorylation of this relevant phosphosite. Interestingly, AAV-mediated overexpression of Rph3A was sufficient to decrease the levels of GluA1 phosphorylated at Ser845 (pSer845-GluA1) in the postsynaptic fraction (TIF), thus suggesting the involvement of phosphorylation processes in the altered distribution of GluA1-containing AMPARs (*n* = 9–8 cultures, *p* < 0.05; Figure 5F,G). However, in AAV-mediated overexpression of Rph3A, only a non-significant decrease in total GluA1 protein levels was detected by WB analysis in the TIF (*n* = 8–9 cultures, *p* = 0.2319; Figure 5F,H). Finally, no significant modification of the pGluA1/GluA1 ratio was detected by WB analysis (*p* = 0.90, Figure 5F,I).

### 3.5. Rph3A Overexpression Occludes cLTP-Dependent Formation of New Spines but Does Not Affect cLTD-Dependent Spine Loss 

**Figure 6:**
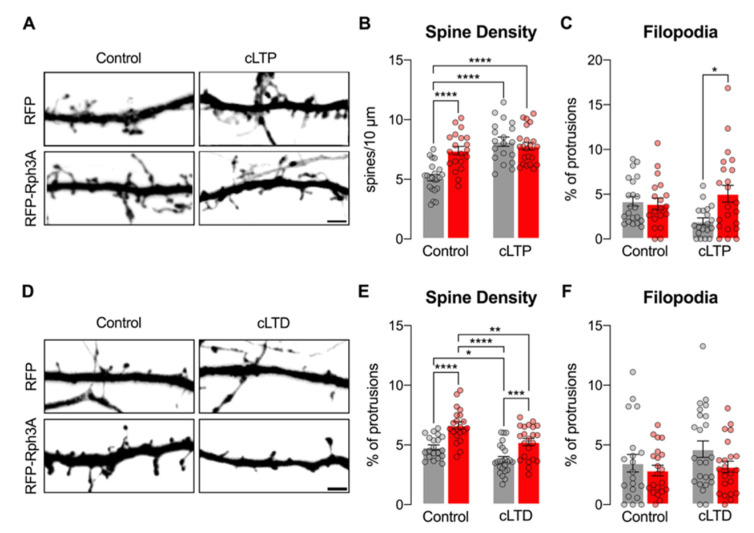
Rph3A affects cLTP but not cLTD. (**A**): Confocal images of GFP filler in the dendritic spines of RFP (**up**) or RFP-Rph3A (**down**) transfected neurons under control conditions (**left**) or after cLTP (**right**). Scale bar: 2 µm. (**B**,**C**): Bar graph representation of mean ± SEM spine density (**B**) and mean ± SEM filopodia proportions (**C**) in RFP (grey) or RFP-Rph3A (red) transfected neurons under control conditions or after cLTP. One-way ANOVA with Tukey post-hoc test. (**D**): Confocal images of GFP filler in the dendritic spines of RFP (**up**) or RFP-Rph3A (**down**) transfected neurons under control conditions (**left**) or after cLTD (**right**). Scale bar: 2 µm. (**E**,**F**): Bar graph representation of mean ± SEM spine density (**E**) and mean ± SEM filopodia proportions (**F**) in RFP (grey) or RFP-Rph3A (red) transfected neurons under control conditions or after cLTD. One-way ANOVA with Tukey post-hoc test. * *p* < 0.05; ** *p* < 0.01; *** *p* < 0.001; **** *p* < 0.0001. Dots represent single values.

It is well known that the induction of LTP and LTD induce bidirectional modifications of dendritic spine density, leading to spine formation and spine loss, respectively [1]. The activation of both AMPARs and NMDARs plays a key role in driving these morphological modifications [30,32]. To test whether the effects of Rph3A overexpression on dendritic spine density (Figure 2), NMDARs (Figure 4) and AMPARs (Figure 5) may affect morphological events associated with synaptic plasticity, we assessed dendritic spine density after the induction of cLTP or cLTD. As expected, cLTP was able to increase dendritic spine density by around 50% in DIV16 neurons transfected with RFP (Figure 6A,B, *n* = 19–22 neurons; *p* < 0.0001; RFP CTRL vs. RFP cLTP). According to the above-described results (see Figure 2), Rph3A overexpression augmented dendritic spine density in resting states compared to RFP control neurons (Figure 6A,B; *n* = 22–20 neurons; *p* < 0.0001; RFP-Rph3A CTRL vs. RFP CTRL). Of note, cLTP was not able to induce any modification of dendritic spine density in RFP-Rph3A neurons (Figure 6A,B; RFP-Rph3A CTRL vs. RFP-Rph3A cLTP; *n* = 20–22 neurons), but promoted only an increase in the proportion of filopodia compared to control neurons (Figure 6C; *n* = 19–22 neurons; *p* < 0.05). We then investigated whether Rph3A overexpression could affect dendritic spine stability upon the induction of cLTD. As expected [22], the induction of cLTD in primary cultures transfected with RFP reduced spine density (Figure 6D,E; *n* = 20–24 neurons; *p* < 0.05; RFP CTRL vs. RFP cLTD). When overexpressing Rph3A, neurons displayed increased levels of spine density compared to RFP transfected cells (Figure 6D,E; *n* = 20–21 neurons; *p* < 0.0001; RFP CTRL vs. RFPRph3A CTRL). However, cLTD induction was still able to significantly decrease spine density (*n* = 21–22 neurons; *p* < 0.01; Figure 6D,E; RFP-Rph3A CTRL vs. RFP-Rph3A cLTD) in a similar percentage as reported for RFP transfected neurons. No differences were observed in the percentage of filopodia (Figure 6F).

**Figure 7:**
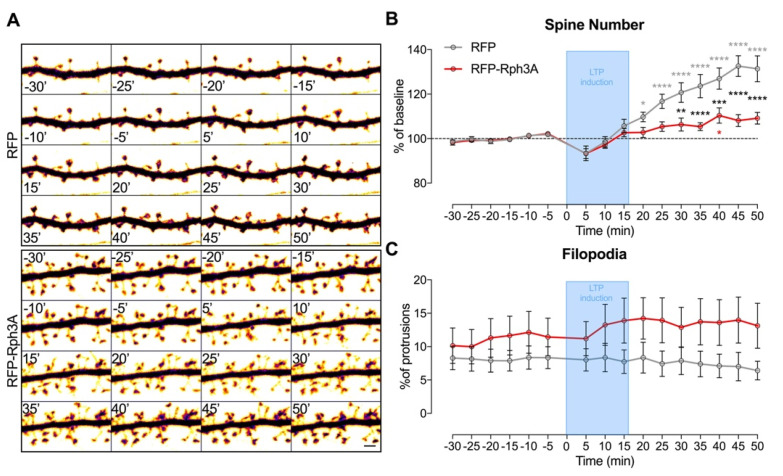
Rph3A overexpression and cLTP in live-imaging of dendritic spines. (**A**): Confocal time lapse images of GFP filler in dendritic spines of RFP (**up**) or RFP-Rph3A (**down**) transfected neurons. Scale bar: 2 µm. (**B**): XY graph representing mean ± SEM spine number as a percentage of T_0_ (0 min) in RFP (grey) or RFP-Rph3A (red) transfected neurons over time before and after cLTP. (**C**): XY graph representing mean ± SEM filopodia as a percentage of total protrusions in RFP (grey) or RFP-Rph3A (red) transfected neurons over time. Repeated measures two-way ANOVA with Dunnett’s multiple comparison test (−15 min vs. all other time points) or Sidak’s multiple comparison test (RFP vs. RFP-Rph3A). * *p* < 0.05, ** *p* < 0.01, *** *p* < 0.001, **** *p* < 0.0001.

Overall, these results highlight that the induced increase in spine density by overexpression of Rph3A is sufficient to prevent the further formation of new spines following cLTP induction. Conversely, Rph3A overexpression did not modify cLTD induced spine loss. To address in more detail the effects of Rph3A overexpression on cLTP induced morphological modifications, the experiments were repeated in a live-imaging confocal setting. As shown in Figure 7A-B, the induction of cLTP in RFP control neurons induced a significant increase in dendritic spine density starting at +20 min compared to the resting state (−15 min), and was significant at all subsequent time points (*n* = 11 neurons; *p* < 0.0001; RFP resting vs. RFP cLTP). However, transfection of RFP-Rph3A almost completely prevented cLTP-induced spine formation and a significant increase in spine density was observed only at one time point (40 min; Figure 7A,B; *n* = 10 neurons; *p* < 0.05; RFP-Rph3A resting vs. RFP-Rph3A cLTP). Importantly, a significant alteration in the dendritic spine increase was observed in RFP-Rph3A neurons compared to RFP neurons starting from 30 min after cLTP induction (Figure 7B; *n* = 11–10 neurons; *p* < 0.0001; RFP-Rph3A vs. RFP), thus confirming a different morphological modification observed in neurons overexpressing Rph3A after the induction of cLTP. No statistically significant difference was observed in the percentage of filopodia over time (Figure 7C).

An analysis of SEP-GluA1 fluorescence at the single spine level was performed to evaluate whether the impaired modifications of dendritic spines in neurons overexpressing Rph3A are correlated with an altered insertion of AMPARs at synaptic membranes. 

**Figure 8:**
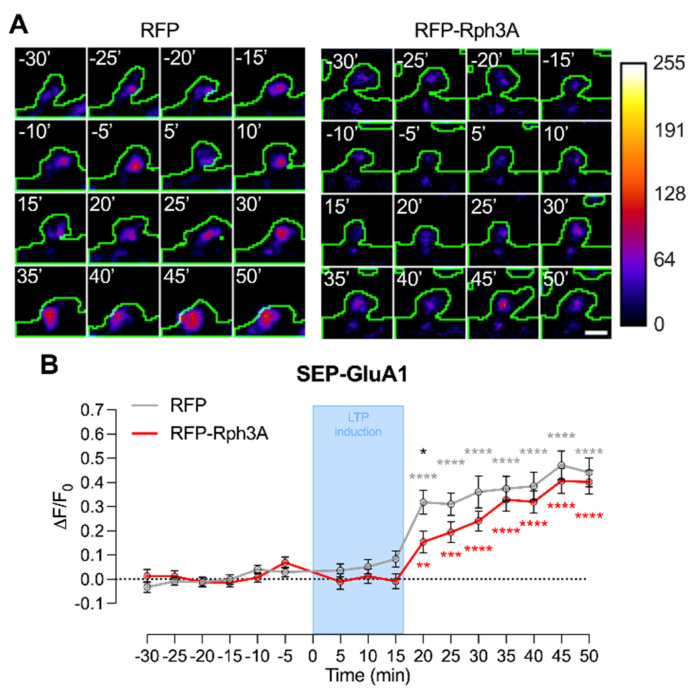
Rph3A overexpression and cLTP in live-imaging of SEP-GluA1 at dendritic spines. (**A**): Confocal time lapse images of SEP-GluA1 in dendritic spines of RFP (**left**) or RFP-Rph3A (**right**) transfected neurons (green outline). Scale bar: 1 µm. (**B**): Graph representing mean ± SEM ΔF/F_0_ of SEP-GluA1 in RFP (grey) or RFP-Rph3A (red) transfected neurons over time before and after cLTP. Repeated measures two-way ANOVA with Dunnett’s multiple comparison test (−20 min vs. other time points, RFP: *grey, RFP-Rph3A: *red) or Sidak’s multiple comparison test (RFP vs. RFP-Rph3A, *black), * *p* < 0.05; ** *p* < 0.01; *** *p* < 0.001; **** *p* < 0.0001.

As shown in Figure 8A,B, though the increase of SEP-GluA1 at the cell surface was seen in both conditions after cLTP, Rph3A transfected neurons are characterized by a slower increase of SEP-GluA1 accumulation at the cell surface compared to control neurons, leading to a significant decrease in surface GluA1 20 min after cLTP induction (Figure 8B; *n* = 90 spines; *p* < 0.05; RFP-Rph3A vs. RFP). No modifications were observed at longer time points (Figure 8B; *n* = 90 spines; RFP-Rph3A vs. RFP).

## 4. Discussion

Physiological synaptic transmission at hippocampal glutamatergic synapses is strictly dependent on the correct synaptic localization of NMDARs, mostly containing the GluN2A regulatory subunit [32]. Accordingly, the molecular mechanisms driving NMDAR synaptic stabilization have been widely examined, and several GluN2A interacting partners involved in synaptic plasticity and learning and memory events have been identified and characterized [3,4]. In particular, protein-protein interactions at the GluN2A CTD have been shown to play a key role in LTP induction [4,6]. However, the correlation between NMDAR synaptic stabilization/enrichment and structural synaptic plasticity at the dendritic spine level has still not been properly addressed.

We previously reported that the induction of LTP promotes Rph3A trafficking at synapses and the formation of a ternary complex with GluN2A and PSD-95, thus leading to an increase in GluN2A synaptic retention. Rph3A silencing blocked cLTP-dependent recruitment of GluN2A-containing NMDARs at cell surface and prevented the formation of new spines [6]. In addition, disruption of Rph3A/GluN2A complex fully prevented LTP induction and led to an impairment of spatial memory [6]. In agreement with these observations, here, we show that mimicking Rph3A accumulation at synapses by Rph3A transfection or viral infection is sufficient to occlude the LTP-induced formation of new dendritic spines, thus confirming a key role for the Rph3A/GluN2A pathway in these events.

Electron microscopy studies found that Rph3A+ spines have an increased spine head area and postsynaptic density size, suggesting an increased stability of neuronal transmission in Rph3A+ connections [6]. Here, we confirmed this hypothesis, showing that Rph3A+ spines are characterized by an augmented turnover of protein translation, thus reinforcing the idea that Rph3A can be considered a marker of potentiated/highly active dendritic spines. Accordingly, Rph3A accumulation at all synaptic connections, as obtained by Rph3A transfection/AAV-mediated infection, prevents the neuron from undergoing cLTP-induced structural modifications at synapses, namely the formation of new dendritic spines. Conversely, no data are currently available regarding the putative involvement of Rph3A in the induction of LTD or in LTD-mediated morphological modifications. Here, we observed that, even if Rph3A overexpression leads to a significant increase in spine density, the induction of cLTD leads to spine loss at a similar extent both in control neurons and in neurons transfected with RFP-Rph3A, suggesting that the role played by Rph3A at excitatory synapses is focused on events correlated with synaptic potentiation but not synaptic depression.

The results presented here also suggest that an aberrant synaptic localization of Rph3A could play a role in pathological forms of synaptic plasticity. In agreement with this hypothesis, an excessive accumulation of Rph3A at the postsynaptic membrane, and an enhancement of Rph3A/GluN2A interactions and GluN2A synaptic levels, have been correlated with pathological synaptic plasticity and absence of synaptic depotentiation in models of late stage of Parkinson’s disease and drug-induced dyskinesia [16].

Several previous reports describing Rph3A synaptic functions have focused on its role at the presynaptic terminal, mediated by a direct interaction with Rab3A [7], CASK [8] and synaptotagmin-1 [9], or on its postsynaptic role directly correlated with NMDAR function [5,6,16]. Importantly, here, we show that postsynaptic Rph3A can also form a triple complex with GluA1 and MyoVa, thus enlarging its network of protein–protein interactions at dendritic spines [12,31]. Interestingly, Rph3A overexpression leads to reduced levels of pSer845-GluA1 and, consequently, to an impairment in GluA1 surface levels. Accordingly, we found that the induction of cLTP in neurons overexpressing Rph3A promotes a slower insertion of SEP-GluA1 at synaptic membranes, thus providing a molecular explanation for the defect in the formation of new spines. However, other studies are needed to clarify the detailed molecular mechanisms linking Rph3A availability at synaptic sites to a modulation of AMPAR localization and activity.

Overall, our present data in agreement with previous studies [5,6,16] indicate that physiological levels of Rph3A at postsynaptic membranes are needed for a correct induction of NMDAR-dependent LTP and structural synaptic plasticity at excitatory synapses. Conversely, aberrant levels of Rph3A induce modifications in GluN2A synaptic retention, leading to altered levels of synaptic NMDARs and morphological impairments [4].

## Data Availability

The data that support the findings of this study are available from the corresponding author upon reasonable request.

## Supplementary Materials

The following supporting information can be downloaded at https://www.mdpi.com/article/10.3390/cells11101616/s1: Figure S1. Representative western blotting analysis of Rph3A performed on neuronal lysates of cultures infected with AAV9-hSyn-RFPRph3A-WPRE and its control AAV9-hSyn-RFP-WPRE.
