# Supplementary material for: Rabphilin-3A Drives Structural Modifications of Dendritic Spines Induced by Long-Term Potentiation
Source: Cells. 2022 May 11;11(10):1616. doi: 10.3390/cells11101616 (PMC9139176; doi:10.3390/cells11101616)
Supplement: Supplementary file 1 [file cells-11-01616-s001.zip › cells-1711911-supplementary.pdf]

Supplementary Figure S1

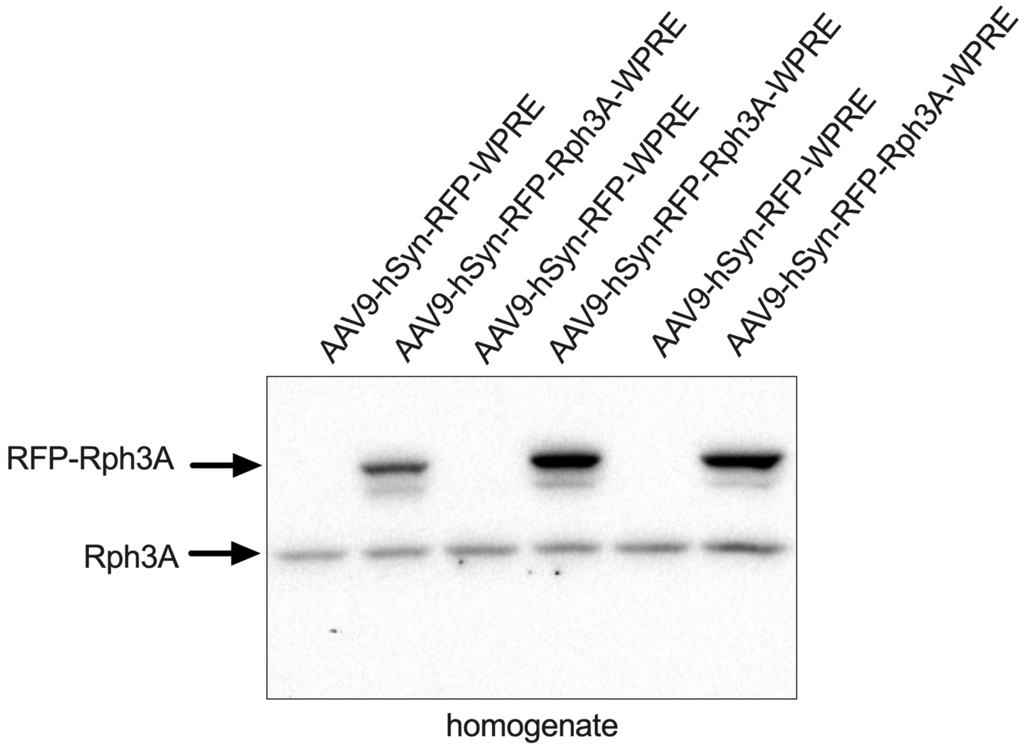

Supplementary Figure S1. Western blotting images of Rph3A from homogenates of hippocampal cultured neurons infected with AAV9-hSyn-RFP-WPRE or AAV9-hSyn-RFPRph3A-WPRE.
